# Effect of Cross-Linking Density on the Structures and Properties of Carbodiimide-Treated Gelatin Matrices as Limbal Stem Cell Niches

**DOI:** 10.3390/ijms19113294

**Published:** 2018-10-23

**Authors:** Jui-Yang Lai, Li-Jyuan Luo, David Hui-Kang Ma

**Affiliations:** 1 Graduate Institute of Biomedical Engineering, Chang Gung University, Taoyuan 33302, Taiwan; judy0028@hotmail.com.tw; 2 Department of Ophthalmology, Chang Gung Memorial Hospital, Linkou, Taoyuan 33305, Taiwan; davidhkma@yahoo.com; 3 Department of Materials Engineering, Ming Chi University of Technology, New Taipei City 24301, Taiwan; 4 Department of Chinese Medicine, Chang Gung University, Taoyuan 33302, Taiwan

**Keywords:** gelatin, carbodiimide, cross-linking density, artificial limbal stem cell niche, corneal epithelial tissue engineering

## Abstract

Given that human amniotic membrane is a valuable biological material not readily available for corneal epithelial tissue engineering, gelatin is considered as a potential alternative to construct a cellular microenvironment. This study investigates, for the first time, the influence of cross-linking density of carbodiimide-treated gelatin matrices on the structures and properties of artificial limbal stem cell niches. Our results showed that an increase in the carbodiimide concentration from 1.5 to 15 mM leads to an upward trend in the structural and suture strength of biopolymers. Furthermore, increasing number of cross-linking bridges capable of linking protein molecules together may reduce their crystallinity. For the samples treated with 50 mM of cross-linker (i.e., the presence of excess *N*-substituted carbodiimide), abundant *N*-acylurea was detected, which was detrimental to the in vitro and in vivo ocular biocompatibility of gelatin matrices. Surface roughness and stiffness of biopolymer substrates were found to be positively correlated with carbodiimide-induced cross-link formation. Significant increases of integrin β1 expression, metabolic activity, and ABCG2 expression were noted as the cross-linker concentration increased, suggesting that the bulk crystalline structure and surface roughness/stiffness of niche attributed to the number of cross-linking bridges may have profound effects on a variety of limbal epithelial cell behaviors, including adhesion, proliferation, and stemness maintenance. In summary, taking the advantages of carbodiimide cross-linking-mediated development of gelatin matrices, new niches with tunable cross-linking densities can provide a significant boost to maintain the limbal stem cells during ex vivo expansion.

## 1. Introduction

In tissue engineering and regenerative medicine, stem cells are powerful and versatile cells that have the ability to differentiate into various mature cell types. Therefore, progress in stem cell research may open new possibilities for reconstructing damaged ocular surfaces and treating various external eye disorders associated with chemical/thermal burn, pterygium, corneal ulcers, Stevens–Johnson’s syndrome, and ocular cicatricial pemphigoid [1]. Several studies have provided compelling evidences for the existence of corneal epithelial stem cells in the limbus [2,3,4,5]. Since limbal epithelium is critical to maintain ocular surface integrity, the potentials of corneal epithelial stem cells for therapeutic applications have gained much attention [6]. In 1997, Pellegrini et al. reported successful restoration of corneal-limbal epithelial defects in patients using autologous cultivated limbal epithelial cell (LEC) sheets [7]. Later, Tsubota et al. transplanted corneal epithelial stem cells from cadaveric eyes into the eyes of patients with severe ocular surface diseases and demonstrated the improvement in visual acuity [8]. Results from these clinical trials suggest that LEC transplantation for corneal tissue repair offers an attractive and promising strategy alternative to lamellar or penetrating keratoplasty.

As an important ophthalmic biomaterial, amniotic membrane displays several advantages: it is a biological tissue derived from human placenta which shows low immunogenicity, and it has anti-inflammatory, anti-angiogenic, and anti-scarring effects that promotes epithelialization and enhances wound healing [9,10]. Investigators have reported that amniotic membrane can be used as a matrix carrier for ex vivo expanded corneal epithelial stem cells as bioengineered ocular surface [11,12,13]. After transplantation of amniotic membrane-limbal epithelial sheet constructs onto the patient’s cornea, a series of manifestations for tissue reconstruction including complete reepithelialization, significant improvement of corneal clarity, and visual acuity are shown at different follow-up stages. Although the application of amniotic membrane seems to be effective in corneal epithelial regenerative medicine, the biological tissue itself is not widely and readily available. In addition, poor standardization related to the human material, risk of donor transmitted infections, and insufficient transparency and strength of matrix carrier are among the main factors affecting the clinical success [14]. It is therefore desirable to develop alternative biomaterials to facilitate ex vivo expansion of transplantable LECs. Increasing efforts have been focused on the fabrication of corneal epithelial equivalents using various culture carriers made of collagen [15], silk fibroin [16], fibrin [17], and keratin [18]. Given that the major portion of the cornea comprises collagenous matrices, these protein-based biomaterials have great application potential in corneal epithelial tissue engineering.

In our laboratory, biological macromolecules such as proteins and polysaccharides have been investigated as ophthalmic biomaterials for tissue engineering [19,20] and drug delivery [21,22]. In particular, gelatin is a denatured form of collagen widely used for food and pharmaceutical industries [23,24]. In comparison with collagen, gelatin is inexpensive and does not express any antigenicity under physiological conditions. Because of its biocompatible and bioadhesive nature, gelatin can be used to exploit a protein-based carrier system for intraocular delivery of corneal endothelial cells [25] and whole retinal tissues [26]. The rapid biodegradation of gelatin carriers following intraocular delivery of cell/tissue sheets may minimize non-specific host inflammatory response, which is beneficial to long-term graft survival and function. However, due to their weak mechanical properties in aqueous media, gelatin materials should be modified to improve their stability so that they can provide a supportive environment for cell attachment and growth. Therefore, porous gelatin hydrogel cross-linked with 1-ethyl-3-(3-dimethyl aminopropyl) carbodiimide hydrochloride (EDC) has also been considered as an alternative to collagen for corneal stromal tissue engineering [27]. These encouraging developments motivate us to explore further use of carbodiimide cross-linked gelatin matrices for the fabrication of limbal stem cell niches.

For limbal stem cell therapy, it is very important to pursue ex vivo cell expansion strategies while retaining the stemness (i.e., undifferentiated state). Biomaterial-based design has proven to be effective for establishing a platform preserving stem cell self-renewal and pluripotent capacity [28]. As reported in the literature, the gelatin matrices possess the ability to support the proliferation of a wide range of stem cell types including embryonic [29], mesenchymal [30], and pluripotent [31] stem cells although little attention has been paid to the role of this biopolymer in limbal stem cell research. Studies of stem cell–material interactions have suggested that the biomaterials can be used to engineer the biophysical and biochemical properties of the extracellular microenvironment, instructing stem cells to adopt a particular cell fate [32,33]. Furthermore, both molecular mobility and surface roughness depending on the cross-linking density of silicon rubber are found to modulate cell growth behaviors in vitro [34]. Given that this study aims to investigate cross-linked gelatin matrices for LEC cultivation, we hypothesize that the cross-linking density of carbodiimide-treated gelatin materials may affect their bulk and surface features to tailor stem cell niches, thereby leading to significant variation in stemness of the LECs. After modification with varying concentrations from 1.5 to 50 mM of EDC, the gelatin substrates were characterized by determinations of cross-linking density, suture strength, and crystalline and chemical structures. Ocular biocompatibility of carbodiimide-treated gelatin matrices as a function of cross-linker concentration was evaluated in vitro using rabbit corneal epithelial cell cultures and in vivo using the anterior chamber of rabbit eyes. The LEC adhesion, proliferation, and stemness in response to matrix topography and stiffness of carbodiimide-treated gelatin were examined to give insight into the effects of cross-linking density of biomaterials on engineering the biophysical cues of the cellular microenvironment niche.

## 2. Results and Discussion

According to our pervious report, carbodiimide cross-linked gelatin carriers exhibit significantly higher molecular stability and greater tissue-encapsulating capability than their non-cross-linked counterparts [35]. Due to its poor mechanical behavior, the native gelatin matrix without chemical modification is prone to rapid dissolution by physiological media, which seriously restricts the use of hydrated gelatin for cell cultivation. Therefore, in this work, non-cross-linked gelatin materials are not considered for the assessment. Since chemically cross-linked gelatin matrix may serve as a niche maintaining the limbal stem cells during ex vivo expansion, it would be interesting to focus on the influence of cross-linking density of protein-based biomaterials in constructing a microenvironment for LEC growth. In order to test the above hypothesis, the study was designed to collect information pertaining to the structure–property–function relationships of gelatin matices of varying extents of cross-linking for corneal epithelial tissue engineering and regenerative medicine.

### 2.1. Cross-Linking of Gelatin Matrices

**Figure 1:**
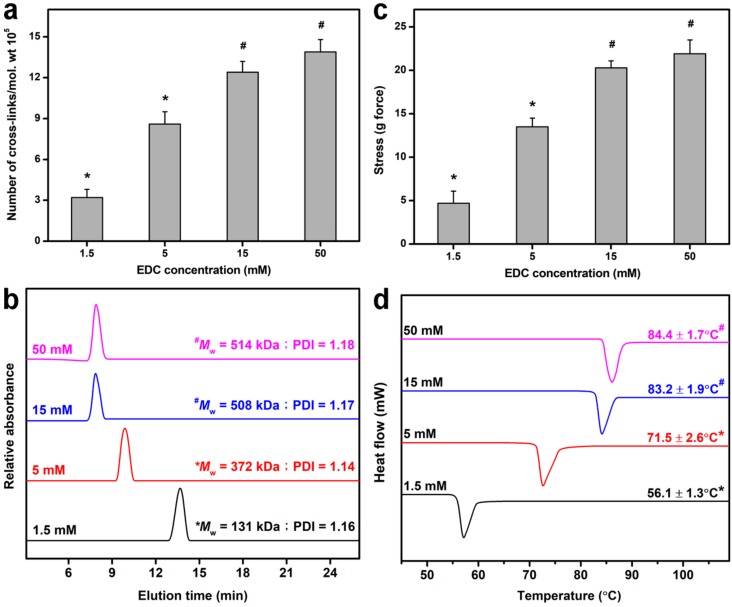
(**a**) Number of cross-links per unit mass, (**b**) weight-average molecular weight, (**c**) suture strength, and (**d**) shrinkage temperature of gelatin samples as a function of carbodiimide concentration. Values are mean ± standard deviation (*n* = 5 for (**a**), *n* = 3 for (**b**), *n* = 6 for (**c**), *n* = 3 for (**d**)). * *p* < 0.05 vs. all groups.

Given that carbodiimide cross-linking of gelatin involves the consumption of free amino groups of lysine residues with accompanying generation of amide linkages (i.e., cross-linking bridges) in protein-based biomaterials (Figure S1) [36], the chemical cross-linking treatment can cause alteration of cross-linked structure of gelatin matrices. The extent of cross-linking and molecular weight (MW) of EDC-treated gelatin was evaluated by cross-linking density measurements (Figure 1a) and gel permeation chromatography (Figure 1b), respectively. Both the number of cross-links per unit mass and weight-average MW were significantly increased with increasing cross-linker concentration from 1.5 to 15 mM (*p* < 0.05). As shown in Figure 1c, an increase in the EDC concentration also led to an upward trend in the gelatin matrix strength. It is known that gelatin is chemically composed of amino acids linked by peptide bonds (i.e., amide linkages). Our results indicate that the structural strength of cross-linked matrices is positively correlated with EDC-induced formation of cross-linking bridges. Furthermore, it has been documented that the averge knot-pull tensile strength of 10-0 nylon suture is 16 g force [37]. In this study, the gelatin materials cross-linked with 15 and 50 mM EDC can meet the strength requirements for surgical suture. Since thermal stability is an important parameter for gelatin-based hydrogels used in biomedical applications [38], the thermal properties of cross-linked gelatin samples were also investigated by differential scanning calorimetry (DSC) (Figure 1d). Here, the suture strength and shrinkage temperature reached a plateau level when the cross-linker concentration was 15 mM. The presence of a larger amount of EDC molecules (i.e., 50 mM) for gelatin stabilization did not further enhance the extent of cross-linking of biomaterials and their resistance to surgical stress and thermal denaturation (*p* > 0.05). It has been documented that the number of carboxylic acid groups within gelatin chains is greater than that of free amino groups available for carbodiimide cross-linking [39]. Therefore, under higher cross-linker concentrations (i.e., above 15 mM), most amino groups are consumed after treatment of gelatin matrices with a relatively large amount of EDC molecules, yielding a similar number of cross-linking bridges.

### 2.2. Structural Characterizations of Cross-Linked Gelatin Matrices

**Figure 2:**
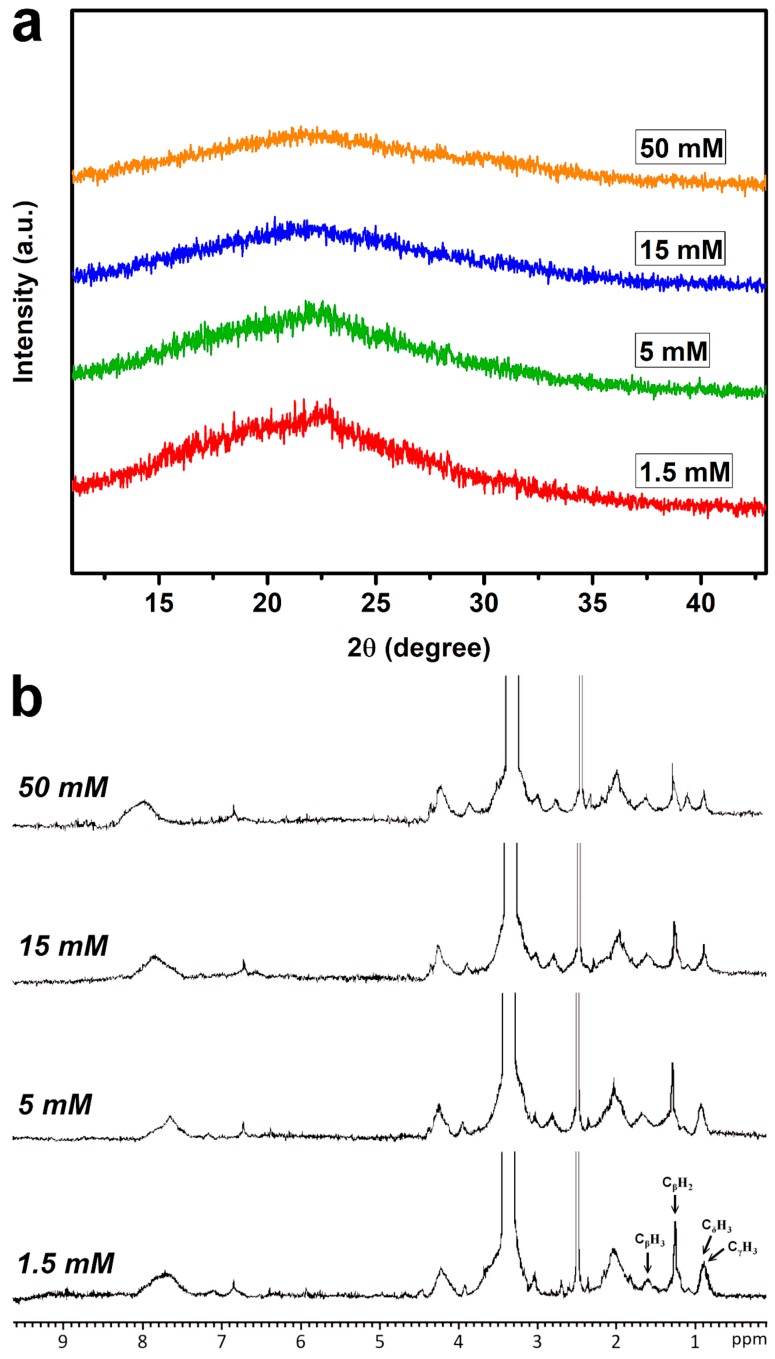
(**a**) XRD patterns and (**b**) ^1^H NMR spectra of various gelatin samples.

Crystallinity is an important bulk structural characteristic of biomaterials influencing the cell behaviors [40]. In this study, the crystalline structure of cross-linked matrices was investigated by XRD measurements. Representative spectra of gelatin samples as a function of EDC concentration are shown in Figure 2a. A broad peak originating from typical triple-helical crystalline structure was present at 2θ value of around 23° in each group [41]. The peak intensity was decreased with increasing cross-linker concentration from 1.5 to 15 mM. In addition, the samples treated with both concentrations of EDC (15 and 50 mM) showed a similar XRD pattern. Overall, the observed variation of crystallinity of cross-linked gelatin matrices is probably due to the variation in the number of cross-linking bridges. The present findings support the report by Manna et al. demonstrating that the increased covalent interaction between gelatin and carboxymethylated guar gum through the formation of amide linkages can significantly reduce the crystallinity of biopolymers [42]. Another possible explanation is that the cross-linking reaction is capable of linking protein molecules together, disturbing crystallization (i.e., the ordered array of molecules) and decreasing crystallinity [43]. On the other hand, it should be noted that the general reaction mechanism of EDC-mediated cross-linking of collagenous biomaterials also involves the binding of carboxylic acid groups with excess *N*-substituted carbodiimide (i.e., an activating agent) to form *N*-acylurea byproducts (Figure S1) [44]. To examine the presence of covalently attached *N*-acylurea groups to the gelatin matrices, NMR spectroscopic analysis was used to characterize the chemical structure of cross-linked materials (Figure 2b). The proton peaks occurring at 1.6, 1.2, and 0.8 ppm were assigned to alanine C_β_H_3_, proline C_β_H_2_, valine C_γ_H_3_, and leucine C_δ_H_3_, respectively, which are typical of those found in gelatin [45]. The signal at 2.6 ppm was attributed to the proton of the solvent. It has been documented that the chemical shift of the methyl triplet of the *N*-ethyl group of EDC in the *N*-acylurea is observed at around 1.1 ppm [46]. An obvious peak referring to structural features of *N*-acylurea was noted only for the samples treated with 50 mM of EDC, indicating that the presence of excess *N*-substituted carbodiimide during material cross-linking reaction contributes to the appearance of this signal. One possible explanation is that the addition of large amounts of EDC to gelatin may increase the collision frequency of remaining carboxylic acid groups of the cross-linked protein molecules and activating agents and thus the formation of abundant *N*-acylurea.

### 2.3. Biocompatibility Assessments of Cross-Linked Gelatin Matrices

**Figure 3:**
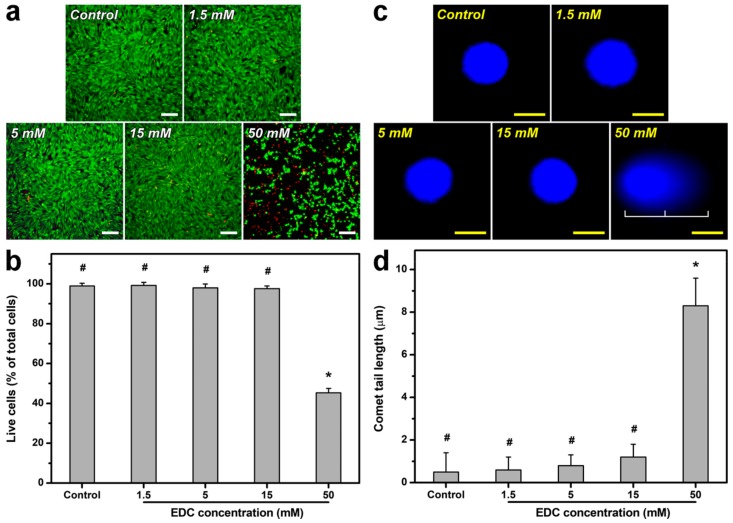
(**a**) Cell viability of SIRC cultures was determined by staining with a live/dead viability/cytotoxicity kit in which live cells fluoresce green and dead cells fluoresce red. Fluorescence images of cells in controls (without materials) after a 3-day exposure to gelatin samples cross-linked with varying concentrations of carbodiimide. (**b**) Mean percentage of live cells in the SIRC cultures exposed to various gelatin samples as measured by the Live/Dead assay. (**c**) Fluorescence photomicrographs and (**d**) comet tail lengths of SIRC cultures exposed to various gelatin samples for 3 days. Control: without test materials. Scale bars: (**a**) 100 μm and (**c**) 10 μm. Values are mean ± standard deviation (*n* = 3 for (**b**), *n* = 4 for (**d**)). * *p* < 0.05 vs. all groups; # *p* < 0.05 vs. 50 mM groups.

It has been well-recognized that biocompatibility is a prerequisite for ophthalmic biomaterials. Given that chemical cross-linking is a process joining molecules via covalent bond formation, the alteration of molecular structures may have effects on cellular and tissue responses to materials. Therefore, the in vitro biocompatibility of carbodiimide cross-linked gelatin matrices was evaluated using a membrane integrity assay on corneal epithelial cells. Figure 3a shows fluorescent images of cells labeled with the Live/Dead stain. After 3 days of incubation in the absence of test materials (i.e., control group), the healthy SIRC cultures exhibited diffuse green fluorescence (indicative of live cells), with very few punctate red-stained nuclei (indicative of dead cells). The cell cultures treated with the gelatin samples cross-linked by EDC at 1.5, 5, and 15 mM had similar fluorescent staining patterns. However, in the test groups exposed to the materials cross-linked with 50 mM of EDC, significantly more red fluorescent spots were found compared to all other groups. As shown in Figure 3b, the SIRC cultures exhibited relatively high levels of cell viability in the control and all test groups (*p* > 0.05), except for those exposed to gelatin matrices cross-linked with 50 mM of EDC. The usage limitation of this specific material is highly associated with its serious cytotoxicity toward rabbit corneal epithelial cultures, as indicated by the results of low mean percentage of live cells. Interestingly, although 15 mM of EDC is sufficient to achieve a plateau in extent of cross-linking, the increase in chemical cross-linker concentration indeed contributes to the differences in molecular structures and interactions in cross-linked gelatin samples, as demonstrated by NMR studies (Figure 2b). Hence, the observed poor cytocompatibility is probably attributed to the existence of covalently attached *N*-acylurea groups to the gelatin matrices treated with 50 mM of EDC. Dong et al. have investigated the effects of urea loading on the growth of human urinary bladder epithelial cells and found that markedly decreased cell viability due to DNA damage and apoptosis occurs when the urea concentration exceeds a critical value [47]. To further elucidate the potential cytotoxicity mechanism of EDC cross-linked gelatin materials carrying abundant *N*-acylurea, the degree of DNA damage in SIRC cells was determined by alkaline comet assays. As shown in Figure 3c, no comet formation was evidenced by intact cell nuclei and smooth margins in the SIRC cultures from the control and all test groups, except for those exposed to gelatin matrices cross-linked with 50 mM of EDC. The mean comet tail length was significantly increased corresponding to an elevated DNA fragmentation level (*p* < 0.05) (Figure 3d), suggesting that the exposure to this specific material may trigger corneal epithelial cell apoptosis and death in vitro. Given that EDC is toxic to cells, it is very important to clarify the issue of biological responses caused by EDC residue. After thorough washing of material samples from various groups with deionized water to remove unreacted EDC, quantitative determination of water-soluble carbodiimides in final wash buffer was performed according to the method reported in the literature involving the use of dimethylbarbituric acid reagent [48]. Our results demonstrate complete removal of unreacted EDC after thorough washing with deionized water. Furthermore, to confirm the toxic effect of unreacted carbodiimide residue due to incomplete removal, the morphology of SIRC cultures was observed after a 3-day exposure to gelatin samples cross-linked with 15 mM of EDC (Figure S2). For the groups of material samples before washing with deionized water, severe cell damage was noted. By contrast, active cell proliferation could be noted in the presence of cross-linked gelatin samples after being thoroughly washed with deionized water to remove unreacted EDC.

**Figure 4:**
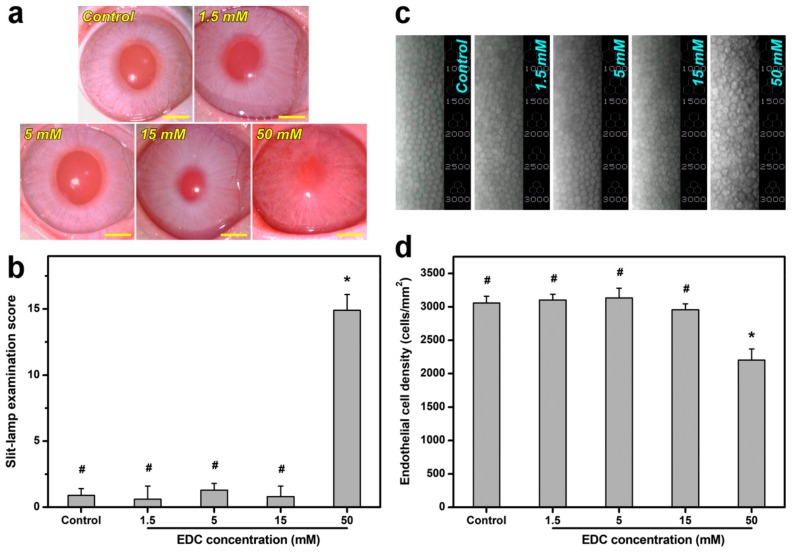
(**a**) Slit-lamp biomicroscopic images and (**b**) examination scores as well as (**c**) specular microscopic images and (**d**) corneal endothelial cell density of rabbit eyes 4 weeks after intracameral implantation of gelatin samples cross-linked with varying concentrations of carbodiimide. Control: sham operation (without test materials). Scale bars: (**a**) 5 mm. Values are mean ± standard deviation (*n* = 6). * *p* < 0.05 vs. all groups; # *p* < 0.05 vs. 50 mM groups.

Here, the in vivo biocompatibility of carbodiimide cross-linked gelatin matrices was also investigated by examining ocular anterior segment tissue reaction. Figure 4a shows slit-lamp biomicroscopic images of rabbit eyes after 28 days of surgical insertion of material implants into the anterior chamber of the eye. In the control (i.e., only corneal/limbal incision without implantation) groups, the sham-operated animal cornea was clear, with no aqueous flare. Similarly, there was no anterior chamber reaction in animal eyes implanted with the gelatin samples cross-linked by EDC at 1.5, 5, and 15 mM. Furthermore, the occurrence of significant loss of material implants in vivo was noted after 4 weeks of biodegradation. In contrast, for the eyes bearing the materials cross-linked with 50 mM of EDC, severe ocular inflammatory responses were evidenced by anterior chamber fibrin, aqueous flare, corneal cloudiness and neovascularization, iris neovascularization, and lens opacity. As shown in Figure 4b, the slit-lamp examination score in the control groups were relatively low, indicating that the sham operation does not cause inflammatory manifestations at 28 days postoperatively. Significantly higher ocular score was found in the test group of gelatin samples cross-linked with 50 mM of EDC than all other groups (*p* < 0.05), suggesting severe tissue responses induced by material implants. Specular microscopic images are also recorded to examine the changes in rabbit corneal endothelial morphology (Figure 4c) and density (Figure 4d). At 28 days postoperatively, the endothelial monolayer showed characteristic hexagonal cell morphology and count in the control and all test groups, except for those exposed to gelatin matrices cross-linked with 50 mM of EDC. Our findings support the aforementioned cytocompatibility studies and demonstrate that the exposure of rabbit corneal endothelium to this specific material leads to abnormal pleomorphic cell shape (i.e., polymorphism) and increased cell size (i.e., polymegathism). It has been documented that the in vivo compensation of cell loss during corneal endothelial monolayer repair mainly depends on the processes of migration and enlargement of neighboring healthy cells [49]. Given that urea is used as a chemical reagent for effective amniotic membrane epithelial cell denudation [10], the corneal endothelium exposed to the EDC cross-linked gelatin materials carrying abundant *N*-acylurea may reasonably undergo cell morphological reorganization. For the first time, here we demonstrate poor in vitro and in vivo ocular biocompatibility is highly correlated with EDC-induced formation of covalently attached *N*-acylurea groups to the gelatin matrices treated with relatively high cross-linker concentration. Based on the consideration that it is not feasible to apply the materials prepared from this specific cross-linking reaction condition, the cultivation of LECs on the gelatin matrices treated with 50 mM of EDC is not performed in later experiments.

### 2.4. Cell Culture Studies on Cross-Linked Gelatin Matrices

For biomedical applications, carbodiimide cross-linked gelatin matrices have been reported over decades [50,51]. Zhang et al. have previously prepared gelatin nanofibers via electrospinning and carbodiimide cross-linking for guided periodontal tissue regeneration [52]. Although the cross-linker concentration is optimized by determining the swelling degree and weight loss of nanofibrous materials, the effect of cross-linking density of gelatin on the periodontal ligament cell behaviors such as attachment, growth, and proliferation is not yet studied. From a corneal tissue engineering perspective, the present work emphasizes the importance of cross-linking density-mediated structures and properties of carbodiimide-treated gelatin matrices in establishing limbal stem cell niches. The morphological features of substrate surfaces are known to be critical for determining the responses of the cultured cells [53]. We have previously examined the surface morphology of chemically cross-linked gelatin hydrogels and found that no visible change in the morphological characteristics can be observed for carbodiimide cross-linked samples compared to the uncross-linked counterparts [54]. Given that EDC is a “zero-length” cross-linking agent without taking part in the linkages, the cross-linking process of gelatin does not significantly alter the morphological properties. However, the carbodiimide-induced formation of cross-linking bridges may lead to the changes in matrix structure and topography, which ultimately affect the cell behaviors. Therefore, this study pays particular attention to the role of bulk crystalline structure and surface roughness/stiffness of the niche attributed to the number of cross-linking bridges in modulating a variety of LEC responses.

#### 2.4.1. Biophysical Cues

**Figure 5:**
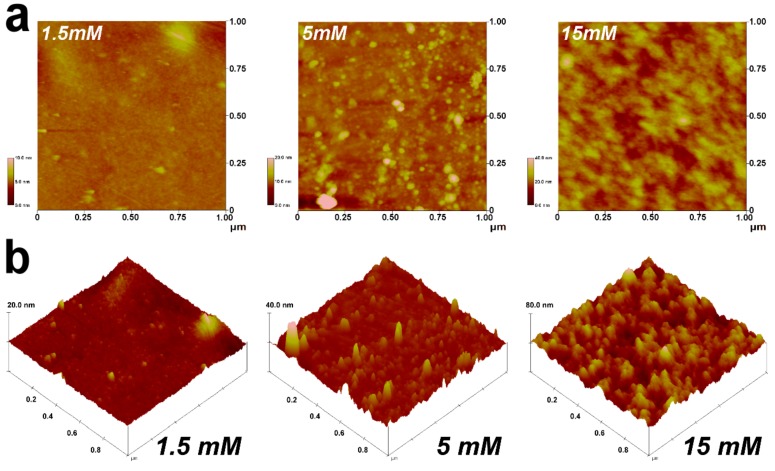

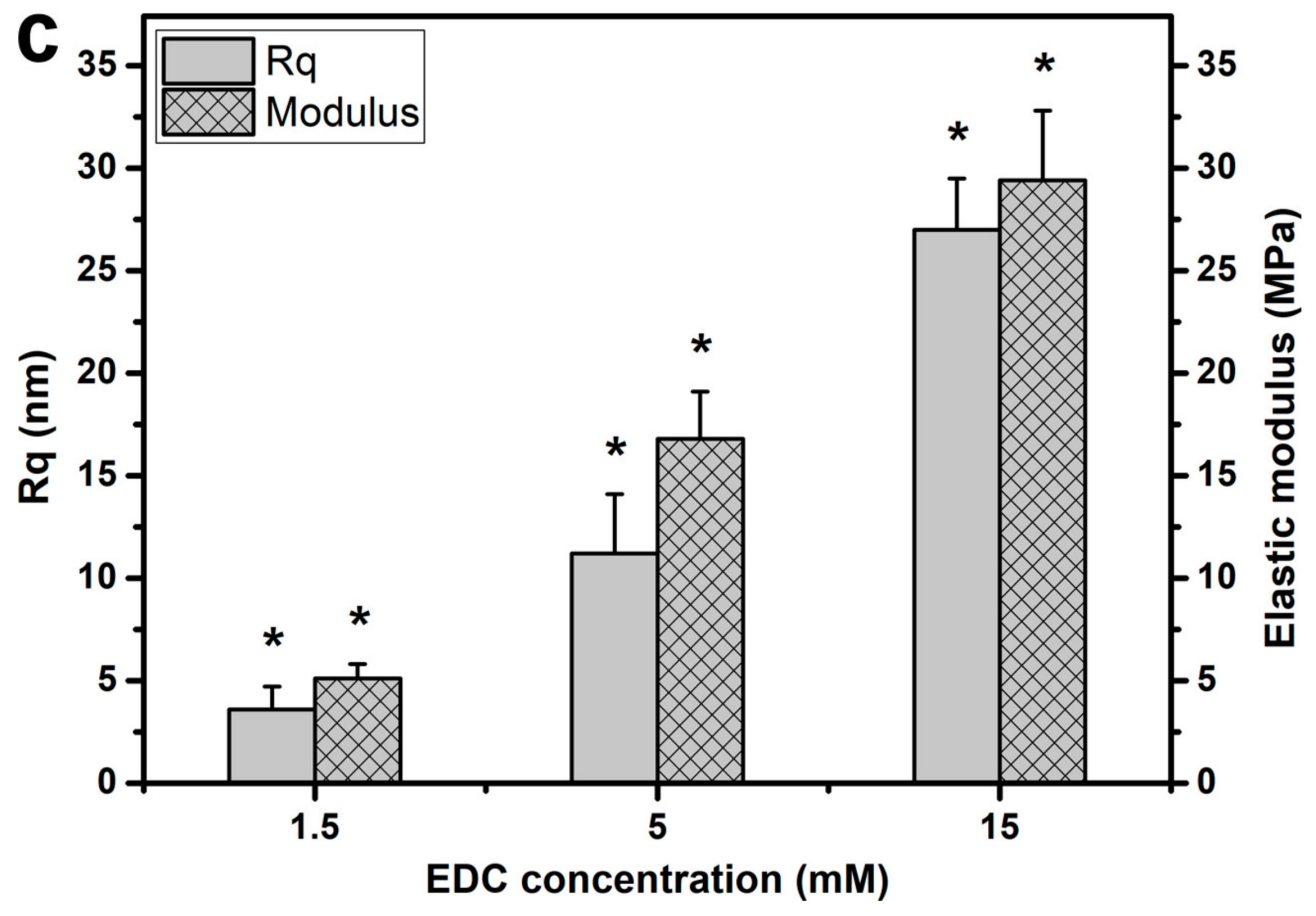
AFM (atomic force microscope) measurements on surfaces of gelatin samples cross-linked with varying concentrations of carbodiimide. (**a**) 2-D and (**b**) 3-D height images; (**c**) Rq (i.e., mean square roughness) and elastic modulus. Values are mean ± standard deviation (*n* = 3). * *p* < 0.05 vs. all groups.

It has been well-recognized that matrix topography and stiffness (i.e., biophysical cues) may provide a crucial set of signals associated with stem cell fate and niche [55]. To clarify the role of cross-linking density of carbodiimide-treated gelatin materials on the biophysical properties of the cellular microenvironment, atomic force microscope (AFM) studies were performed to determine the matrix topography and stiffness. According to the tapping mode AFM height images (Figure 5a,b), the surface for the gelatin samples cross-linked with 1.5 mM EDC appeared generally smooth to finely papillate. However, when the materials treated with EDC at 5 and 15 mM, different surface features were noted, probably due to the increased interaction between protein molecules and chemical cross-linkers. Our data demonstrated that the formation of a higher number of cross-linking bridges between adjacent biopolymer chains may lead to a rougher topography with the existence of more noticeable ridges and bumps. As shown in Figure 5c, both surface roughness and elastic modulus values were significantly increased with increasing cross-linker concentration from 1.5 to 15 mM (*p* < 0.05). The results indicate that chemical modification of gelatin with large amounts of EDC may facilitate the cross-link-induced aggregation of polypeptide by drawing the molecules closer together, thus altering the matrix topography and stiffness. Our findings also reflect the importance of cross-linking density of biomaterials in regulating their surface roughness and stiffness.

#### 2.4.2. Cell Adhesion

**Figure 6:**
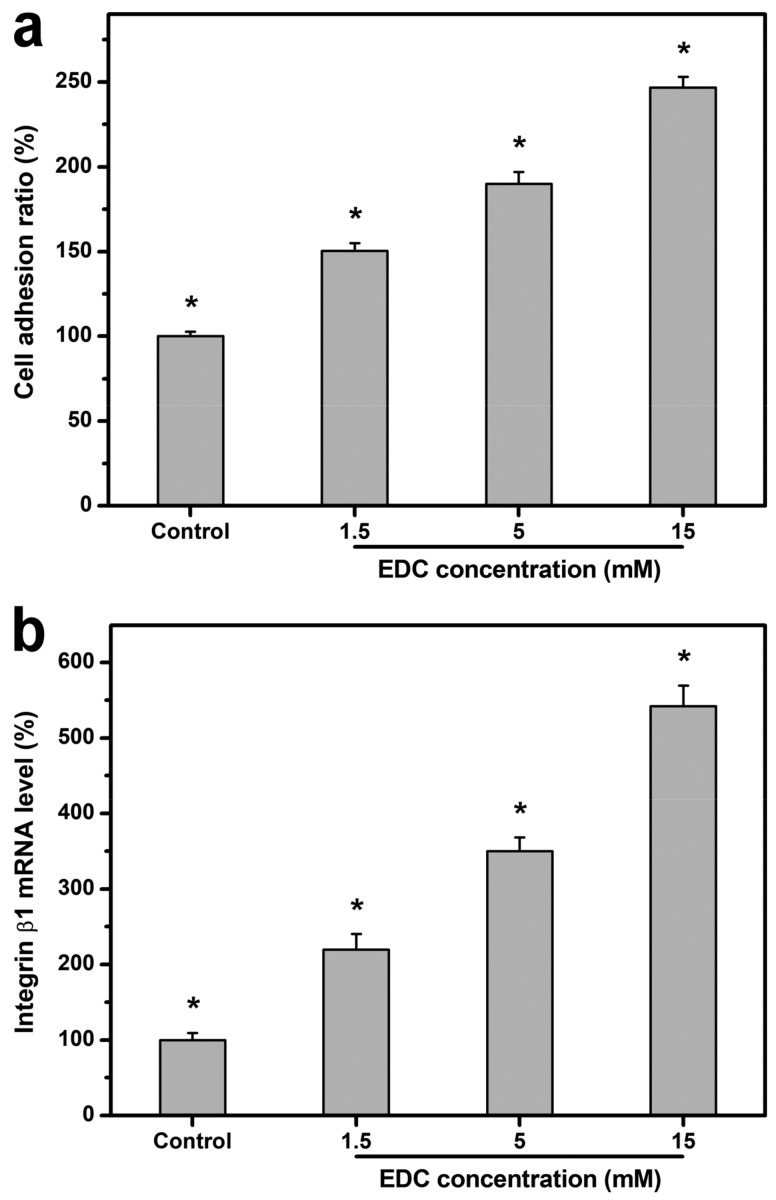
(**a**) Cell adhesion ratio and (**b**) gene expression level of integrin β1 on various gelatin substrates after rabbit LEC (limbal epithelial cell) seeding for 8 h. Normalization was done by using glyceraldehyde-3-phosphate dehydrogenase (GAPDH). Results are expressed as a percentage of control (i.e., cells cultured on TCPS substrates) values. Values are mean ± standard deviation (*n* = 4). * *p* < 0.05 vs. all groups.

Given that biomaterial substrates can provide a template for cell-anchorage growth, it is very crucial to assess the adhesion of LECs to the carbodiimide-treated gelatin matrices with varying numbers of cross-linking bridges. As shown in Figure 6a, the cell adhesion ratio in the control groups (i.e., standard tissue culture polystyrene (TCPS) substrates) was significantly lower than that of all experimental groups (i.e., cross-linked gelatin substrates) (*p* < 0.05), possibly related to promotion of LEC adhesion by gelatin. Furthermore, the cell adhesion ratio was significantly increased with increasing cross-linker concentration from 1.5 to 15 mM (*p* < 0.05). As a well-known matrix molecule, gelatin containing the Arg-Gly-Asp (RGD) sequences is able to support cell adhesion and interact with integrin. Interestingly, although the chemical modification of gelatin with carbodiimide essentially involves consuming aspartic acid residues in protein and reducing the content of RGD during cross-linking reaction, the increase in cross-linking density of biomaterial substrates indeed contributes to the enhanced cell adhesion on gelatin matrices. Our results imply the effect of bulk and surface structural characteristic of cross-linked gelatin on cell attachment to the underlying substratum. When considering the cell–substrate crosstalk, the cell adhesion force is found to be dependent upon the surface roughness [56] and stiffness of biomaterials [57]. In addition, crystallinity is likely a major factor leading to differences in cell adhesion on biomaterials [58]. Uygun et al. have shown that the amount of rat hepatocytes attached to the chitosan membranes increases with decreasing substrate crystallinity [59]. The present findings are compatible with these earlier observations and motivate further studies related to the effects of cross-linking density of biomaterials on the expression of cell–matrix adhesion molecules during the interactions between LECs and gelatin substrates. Therefore, the integrin β1 expression at mRNA level was detected by means of qRT-PCR (Figure 6b). The gene expression level in the control groups (i.e., standard TCPS substrates) was significantly lower than that of all experimental groups (i.e., cross-linked gelatin substrates) (*p* < 0.05). Moreover, significant increases of integrin β1 expressions were noted as the cross-linker concentration during chemical modification of gelatin increased (*p* < 0.05). The experimental results show a similar trend to the adhesion ratio comparison, suggesting that LEC attachment to the substrate materials is positively associated with integrin signal strength.

#### 2.4.3. Cell Proliferation

**Figure 7:**
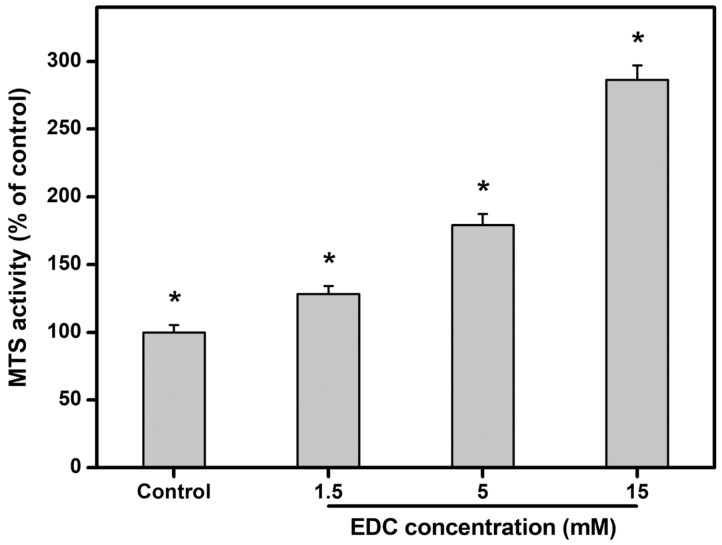
MTS activity (mitochondrial dehydrogenase activity) on various gelatin substrates after rabbit LEC growth for 5 days. Results are expressed as a percentage of control (i.e., cells cultured on TCPS substrates) values. Values are mean ± standard deviation (*n* = 4). * *p* < 0.05 vs. all groups.

In this work, the growth of rabbit LECs on carbodiimide-treated gelatin matrices with varying numbers of cross-linking bridges was assessed by determination of mitochondrial dehydrogenase activity (MTS activity) (Figure 7). After incubation for 5 days, the activity level in the control groups (i.e., standard TCPS substrates) was set to 100%. The MTS activity in the cells expanded on the gelatin substrates cross-linked with 15 mM of EDC was significantly higher than that of the other cross-linked gelatin substrate groups (*p* < 0.05). To exclude the effect of initial cell attachment on cell proliferation behaviors, the amount of proliferated LECs on day 5 in the 1.5, 5, and 15 mM groups was further calculated and compared after normalization to the number of adherent cells at 8 h post-seeding. The MTS activity levels were significantly greater in 15 mM (1.36-fold increase) and 5 mM (1.11-fold increase) groups than those in 1.5 mM groups (*p* < 0.05), indicating that LEC proliferative capacity is independent of better cell attachment at the initial stage. Investigators have previously demonstrated increased rat mesenchymal stem cell proliferation on less crystalline chitosan materials [59]. With increasing hydroxyvalerate content, the crystallinity of poly(3-hydroxybutyrate-*co*-3-hydroxyvalerate) membranes decreases, thereby enhancing the metabolic activity of human mesenchymal stem cells [60]. Our findings support these earlier observations indicating that substrate crystallinity of carbodiimide-treated gelatin matrices may have profound effects on LEC growth regulation. On the other hand, it has been reported that prolonging the time of electrochemical etching of silicon wafers can increase the surface roughnesses that favor the proliferation of human umbilical vein endothelial cells [61]. In addition to the substrate nanotopography, stiffness is found to affect the cell population during ex vivo expansion. Peyton et al. have shown that smooth muscle cells grown on stiffer poly(ethylene glycol) substrates undergo a greater extent of proliferation [62]. Rowlands et al. have further demonstrated that as gel stiffness increases, human mesenchymal stem cell proliferation is promoted by the enhancement of integrin signaling [63]. The results of the present study are in accordance with these previous findings, and indicate that active LEC proliferation is obvious on stiffer matrices attributed to higher cross-linking density of carbodiimide-treated gelatin.

#### 2.4.4. Cell Stemness

**Figure 8:**
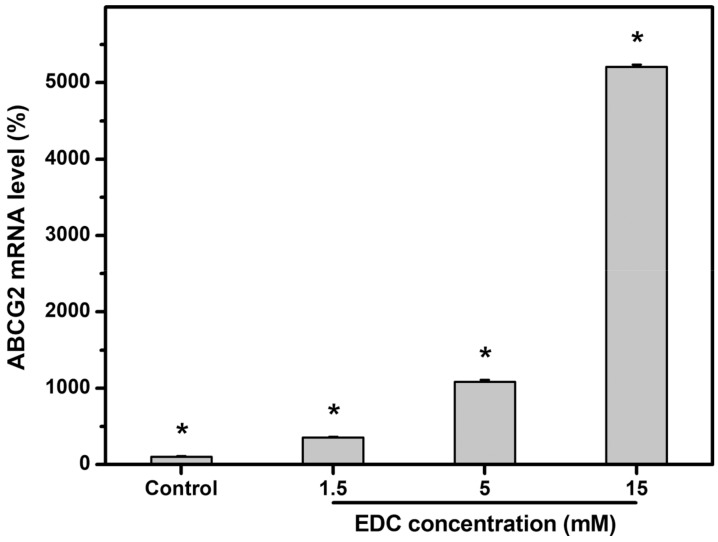
Gene expression level of ABCG2 on various gelatin substrates after rabbit LEC growth for 5 days. Normalization was done by using GAPDH. Results are expressed as a percentage of control (i.e., cells cultured on TCPS substrates) values. Values are mean ± standard deviation (*n* = 4). * *p* < 0.05 vs. all groups.

It is known that the surrounding microenvironment (i.e., niche) influences stem cell fate decisions [64]. Grueterich et al. have reported that the amniotic membrane contains various growth factors, anti-inflammatory proteins, and protease inhibitors, and may serve as a niche for the maintenance of limbal stem cells during ex vivo expansion [65]. Our group has proposed chemically modified amniotic membrane to enhance the mechanical stability and resistance to collagenase digestion while preserving corneal epithelial progenitor cells in vitro [66]. When compared with its native biological tissue counterpart, cross-linked amniotic membrane may represent a promising means to provide an appropriate microenvironment for LEC growth. However, the amniotic membrane is a valuable biological material not readily available. Herein, carbodiimide cross-linked gelatin matrices are considered as candidates for limbal stem cell niches. The stemness of rabbit LECs on carbodiimide-treated gelatin matrices with varying numbers of cross-linking bridges was assessed by determination of the expression of ABCG2 (i.e., limbal epithelial stem cell marker [66]) after incubation for 5 days. As shown in Figure 8, the mRNA level detected by qRT-PCR was defined as 100% in the control groups (i.e., standard TCPS substrates). The relative expression of ABCG2 transcripts were significantly increased with increasing cross-linker concentration from 1.5 to 15 mM (*p* < 0.05). In particular, gene expression reached the highest level on the biomaterial substrates cross-linked with 15 mM, with 480% amplification over the counterparts modified with 5 mM, suggesting enhanced ability of gelatin matrices with higher cross-linking density to preserve corneal epithelial progenitor cells in vitro. Given that the regulation of niche properties is crucial to maintain cells in an undifferentiated state (indicative of stemness), it is essential to explore the bulk and surface structural characteristic of cross-linked gelatin responsible for mediating cell differentiation. When compared with its crystalline coating counterpart, amorphous calcium phosphate coating material is found to be unfavorable for osteoblast differentiation of rat bone marrow-derived mesenchymal stem cells [67]. A previous study has also shown that stimulations from a stiffer matrix decrease the degree of smooth muscle cell differentiation [62]. Marletta et al. have investigated the effects of surface roughness of polyester films on osteoblastic induction of human marrow stromal cells and found that increasing nanotopographical roughness inhibits cell differentiation [68]. Since the rougher surface of biomaterial substrates can theoretically have more contact area, it is reasonable to speculate that more topographical cues for recognition by integrins and maintenance of cell undifferentiation in our case of using gelatin matrices modified with higher concentration of EDC. Overall, the results of the present study suggest that the expression of LEC stemness on carbodiimide-treated gelatin matrices is perhaps related to the crystalline structure (Figure 2a) and surface roughness/stiffness (Figure 5c) of niche as a result of varying cross-linking densities (Figure 1a). In addition, cells maintaining stemness should possess an active proliferating capacity, and better cell attachment may lead to higher proliferation. On the other hand, it is interesting to note that although carbodiimide treatment is applied to both natural-origin biomaterials, the cross-linked gelatin may have a better ability to preserve corneal epithelial progenitor cells than chemically modified amniotic membrane [69]. Nevertheless, the mechanism of cell stemness maintenance attributed to substrate material nature remains unclear. Our future interest will be toward establishing a carbodiimide cross-linked gelatin matrices-based platform for better regulation of stem cell proliferation and differentiation.

## 3. Materials and Methods

### 3.1. Materials

Gelatin (type A; 300 Bloom) and 1-ethyl-3-(3-dimethyl aminopropyl) carbodiimide hydrochloride (EDC) were obtained from Sigma-Aldrich (St. Louis, MO, USA). Eagle’s minimum essential medium (MEM), trypsin-ethylenediaminetetraacetic acid (EDTA), Dulbecco’s modified Eagle’s medium (DMEM), Ham’s F-12 nutrient mixture (Ham’s F-12), gentamicin, and TRIzol reagent were purchased from Gibco-BRL (Grand Island, NY, USA). Fetal bovine serum (FBS) was acquired from Biological Industries (Kibbutz Beit Haemek, Israel). Live/Dead Viability/Cytotoxicity Kit was purchased from Molecular Probes (Eugene, OR, USA). 4′,6-diamidino-2-phenylindole (DAPI) was acquired from Vector Laboratories (Peterborough, England). Tiletamine hydrochloride/zolazepam hydrochloride mixture and xylazine hydrochloride were obtained from Virbac (Carros, France) and Bayer (Leverkusen, Germany), respectively. Proparacaine hydrochloride ophthalmic solution was acquired from Alcon-Couvreur (Puurs, Belgium). Atropine sulfate ophthalmic solution was obtained from Oasis (Taipei, Taiwan). Dispase II was acquired from Roche Diagnostics (Indianapolis, IN, USA). Dimethyl sulfoxide (DMSO) was obtained from J.T.Baker (Phillipsburg, NJ, USA). 24-well tissue culture polystyrene (TCPS) plate was purchased from Becton Dickinson Labware (Franklin Lakes, NJ, USA). CellTiter 96 Aqueous One Solution Cell Proliferation Assay Kit was purchased from Promega (Madison, WI, USA).

### 3.2. Cross-Linking of Gelatin Matrices

To prepare cross-linked material samples, an aqueous solution of 10 wt% gelatin was first cast into a polystyrene planar mold and air-dried at 25 °C for 24 h. The gelatin sheets were further treated by carbodiimide using a film immersion method [35]. After incubation in an ethanol/water mixture (8:2, *v*/*v*, pH 4.75) of 0-50 mM EDC, the material samples were cross-linked at 25 °C for 24 h. Then, the gelatin sheets were thoroughly washed with deionized water to remove unreacted EDC and further dried in vacuo.

The cross-linking density was evaluated on the basis of the theory of rubber elasticity [70]. After immersion in deionized water at the testing temperature (i.e., 25 °C), the gelatin hydrogel sheets (20 mm × 10 mm) were mounted between two clamps of an Instron Mini 44 universal testing machine (Canton, MA, USA) for measurement of mechanical properties and stress–strain curves. Following the removal of the specimens from the clamps, the density was measured using specific gravity bottle method. A graph of *σ* against (*α*−*α*^−2^) would be a straight line with the slope giving *RTρV*^1/3^/*M*_c_, where *σ* = the force per unit area of the swollen unstretched sample; *α* = extension ratio; *R* = gas constant; *T* = absolute temperature; *ρ* = density of specimen; *V* = volume fraction; and *M*_c_ = average molecular weight of the chains between cross-links. The number of cross-links per unit mass would be given by (2*M*_c_)^−1^. Results were averaged on five independent runs.

To determine the weight-average MW (*M*_w_) and polydispersity index (PDI) of various cross-linked gelatin samples, gel permeation chromatography was performed according to our previously published method [54]. The system for gel permeation chromatography consisted of a HPLC-pump and a RI 2000 refractive index detector (Schambeck SFD GmbH, Bad Honnef, Germany) with four thermostated (37 °C) columns (Shodex SB series, OHpak SB-802 HQ (exclusion limit 1 × 10^3^ Da); OHpak SB-802.5 HQ (exclusion limit 1 × 10^4^ Da); OHpak SB-803 HQ (exclusion limit 1 × 10^5^ Da); OHpak SB-804 HQ (exclusion limit 1 × 10^6^ Da); Showa Denko, Tokyo, Japan). Aqueous solution of 62.5 mM of sodium dodecyl sulfate was used as the eluent at a flow rate of 1 mL/min. The chromatograms were analyzed with Analab EC2000 Data System to calculate MW based on a calibration curve recorded with narrow MW distribution poly(ethylene glycol) standards (Polymer Standards Service, Mainz, Germany). Results were averaged on three independent runs.

The suture strength of gelatin sheets was determined by suture pull out tests [71]. The fully swollen samples were suspended between two diametrically positioned 10-0 nylon sutures, penetrating through the hydrogels at 2 mm from the edge. The free ends of each suture were mounted between two clamps of an Instron Mini 44 universal testing machine. The maximum load at break was measured at a crosshead speed of 5 mm/min. Results were averaged on six independent runs.

### 3.3. Structural Characterizations of Cross-Linked Gelatin Matrices

X-ray spectra of various gelatin samples were recorded using a Bruker AXS D8 Advance X-ray diffractometer (Karksruhe, Germany) under Cu Kα radiation operated at 40 kV and 30 mA. After placement of test materials on a sample stage, the X-ray diffraction (XRD) patterns were collected over a 2θ scan range of 11–43° with a rate of 1°/min and a step size of 0.3°. On the other hand, the gelatin samples were characterized by hydrogen-1 nuclear magnetic (^1^H NMR) spectroscopy. Proton NMR spectra were recorded at 500 MHz using a Bruker Avance DRX 500 NMR instrument (Taipei Medical University, Taipei, Taiwan). The ^1^H chemical shift scale was referenced against internal DMSO-d6 at 2.6 ppm.

### 3.4. Biocompatibility Assessments of Cross-Linked Gelatin Matrices

SIRC cells, a rabbit corneal epithelial cell line (BCRC no. 60093), were obtained from the Bioresource Collection and Research Center (Hsinchu, Taiwan). The cells were maintained in regular growth medium containing MEM and 10% FBS, and cultured in a humidified atmosphere of 5% CO_2_ at 37 °C. In this study, the cells from passage 425 were used for in vitro biocompatibility tests. SIRC cells with a density of 5 × 10^4^ cells/mL were seeded in 24-well plates by 1 mL/well. After sterilization in a 70% ethanol solution, the gelatin samples (200 mg) were added to the regular growth medium and incubated for 3 days. Cell cultures without contacting with the material samples served as control groups. To examine the SIRC cells exposed to the cross-linked gelatin, the cell viability was evaluated using a membrane integrity assay [19]. After washing three times with phosphate-buffered saline, the cultures were stained with the Live/Dead Viability/Cytotoxicity Kit containing calcein acetoxymethyl and ethidium homodimer-1 to identify the live and dead cells, respectively. Under fluorescence microscopy (Carl Zeiss, Oberkochen, Germany), the number of live and dead cells was counted. All experiments were performed in triplicate, and the viability was expressed as the average ratio of live cells to the total number of cells. The degree of DNA damage of SIRC cells was also measured using the comet assay [22]. After cell lysis and electrophoresis, nuclei were stained with DAPI for comet visualization under a fluorescence microscope (Carl Zeiss). Quantification of the DNA damage for each cell was made by calculating as comet tail length (μm) = maximum total length—head diameter. Results were the average of four independent measurements.

For in vivo biocompatibility studies, thirty New Zealand white rabbits (National Laboratory Animal Breeding and Research Center, Taipei, Taiwan) weighing 2.5–3.0 kg were used as experimental animals. All animal procedures were performed in accordance with the ARVO Statement for the Use of Animals in Ophthalmic and Vision Research. The animal use protocols were approved by the Institutional Animal Care and Use Committee of Chang Gung University (CGU14-074, 7 March 2015). In the four test groups (1.5, 5, 15, and 50 mM) of animals (six rabbits/group), the ethanol-sterilized gelatin implants (7-mm-diameter) were surgically placed into rabbit ocular anterior chamber according to the procedure described elsewhere [36]. The remaining six rabbits received no implant (only corneal/limbal incision) and served as a control group. After intramuscular anesthesia with tiletamine hydrochloride/zolazepam hydrochloride mixture (2.5 mg/kg) and xylazine hydrochloride (1 mg/kg), the rabbits were topically instilled with two drops of proparacaine hydrochloride ophthalmic solution (0.5%) and one drop of atropine sulfate ophthalmic solution (1%). Under the surgical microscope (Carl Zeiss), a 7.5-mm corneal/limbal incision was made to allow sample implantation into the anterior chamber. The incision site was closed with 10-0 nylon sutures. The implant–tissue interaction was determined by performing diagnostic measurements on ocular anterior segment at 28 days postoperatively. By means of slit-lamp biomicroscopy (Topcon Optical, Tokyo, Japan), the anterior chamber activity, corneal and lens clarity, and iris neovascularization were assessed. The ocular grading method used for biomicroscopic examinations is shown in Table 1. During clinical assessment, six ophthalmic parameters were recorded from animal eyes and were numerically graded on an increasing severity scale of 0–4. For each parameter, mean ocular score was calculated as the sum of the scores divided by the number of eyes in each group. Overall ocular score was expressed as the summation of six mean scores in the same studied group. Furthermore, the corneal endothelial cell density in the rabbit eyes at postoperative day 28 was determined by specular microscopy (Topcon Optical). Each data point is an average of three independent observations during the measurements.

### 3.5. Cell Culture Studies on Cross-Linked Gelatin Matrices

An atomic force microscope (AFM) (NanoScope IV; Veeco Digital Instruments, Santa Barbara, CA, USA) was utilized to scan surface topography of the gelatin substrates. All measurements were made in tapping mode using a silicon cantilever at room temperature. AFM images were recorded with a scan size of 1 μm × 1 μm. Five measurements were done on different surface sites to calculate the root mean square roughness (Rq) for each sample. Results were averaged on three independent runs. The nanoscale mechanical properties of the gelatin samples were analyzed by force-volume spectroscopy using the same AFM. A silicon cantilever operated in contact mode was employed. During the measurements, a defined force was applied to various surface sites using a calibrated tip. The amount of cantilever deflection was monitored to determine the surface elastic modulus. Results were the average of three independent measurements.

For in vitro cell culture studies, twenty New Zealand white rabbits (National Laboratory Animal Breeding and Research Center, Taipei, Taiwan) weighing 2.5–3.0 kg were used as experimental animals. All animal procedures were performed in accordance with the ARVO Statement for the Use of Animals in Ophthalmic and Vision Research and approved by the Institutional Animal Care and Use Committee of Chang Gung University (CGU14-074, 7 March 2015). To disperse the cells, the rabbit corneoscleral rims were treated with dispase II at 37 °C, followed by incubation with trypsin-EDTA solution. The LEC cultures were maintained with supplemental hormonal epithelial medium (SHEM), which was made of an equal volume of HEPES-buffered DMEM containing bicarbonate and Ham’s F-12, 0.5% DMSO, 2 ng/mL mouse epidermal growth factor, 5 μg/mL insulin, 5 μg/mL transferrin, 5 ng/mL selenium, 0.5 μg/mL hydrocortisone, 30 ng/mL cholera toxin A subunit, 5% FBS, 50 μg/mL gentamicin, and 1.25 μg/mL amphotericin B.

Rabbit LECs with a density of 5 × 10^4^ cells/mL were seeded into 24-well TCPS plates (control groups) and various sterilized gelatin substrates by 1 mL/well, and incubated at 37 °C for 8 h. The number of LECs attached to the material samples was determined by the CellTiter 96 Aqueous Non-Radioactive Cell Proliferation MTS Assay, in which MTS tetrazolium compound is bio-reduced by cells to form a water-soluble colored formazan. After incubation of cell cultures with MTS/PMS (20:1) reagent, the data of absorbance readings at 490 nm were measured using the Multiskan Spectrum Microplate Spectrophotometer (ThermoLabsystems, Vantaa, Finland). All experiments were carried out in quadruplicate, and the results were expressed as relative cell adhesion ratio compared to the controls (TCPS groups). Furthermore, the total RNA was isolated from cells with TRIzol reagent according to the manufacturer’s procedure. Reverse transcription of the extracted RNA (1 μg) was performed using ImProm-II (Promega) and Oligo(dT)_15_ primers (Promega). The sequences of the primer pairs for rabbit integrin β1 are listed in Table 2. Quantitative real-time reverse transcription polymerase chain reaction (RT-PCR) was performed on a Light-Cycler instrument (Roche Diagnostics) according to the manufacturer’s instructions with FastStart DNA Master SYBR Green I reagent. Each sample was determined in quadruplicate, and the gene expression results were normalized to the level of glyceraldehyde-3-phosphate dehydrogenase (GAPDH) mRNA.

Cell proliferation was further evaluated by attaching the same number of rabbit LECs (3 × 10^4^ cells/test material) to various sterilized gelatin substrates, and incubated at 37 °C for 5 days. The LECs cultured on TCPS served as control groups. The proliferation of the cells was measured using the CellTiter 96 Aqueous Non-Radioactive Cell Proliferation MTS Assay again. Results were averaged on four independent runs and expressed as relative MTS activity compared to the controls. The total RNA was also isolated from LECs for the analysis of ABCG2 expression level. The sequences of the primer pairs for rabbit ABCG2 are listed in Table 2. Gene expression results were averaged on four independent runs and normalized to the level of GAPDH mRNA.

### 3.6. Statistical Analyses

Results were expressed as mean ± standard deviation (SD). Significance was evaluated by one-way analysis of variance (ANOVA) and accepted with *p* < 0.05.

## 4. Conclusions

In the field of tissue engineering, cross-linked gelatin matrices have been widely described for cultivation of mesenchymal stem cells from different sources [72,73,74]. To the best of our knowledge, the influence of cross-linking density of carbodiimide-treated gelatin matrices on the structures and properties of limbal stem cell niches has not been investigated yet. Our results show that an increase in the EDC concentration from 1.5 to 15 mM leads to an upward trend in the structural and suture strength of gelatin matrices, thereby reducing the crystallinity of biopolymers. For the samples treated with 50 mM of EDC, the presence of excess *N*-substituted carbodiimide during the material cross-linking reaction contributes to the formation of abundant *N*-acylurea, which is detrimental to in vitro and in vivo ocular biocompatibility of gelatin matrices. Surface roughness and stiffness of gelatin substrates are found to be positively correlated with EDC-induced cross-link formation. Significant increases of integrin β1 expression, metabolic activity, and ABCG2 expression are noted as the cross-linker concentration during chemical modification of gelatin increases, suggesting that the crystalline structure and surface roughness/stiffness of niche attributed to the number of cross-linking bridges may have profound effects on a variety of cellular behaviors, including LEC adhesion, proliferation, and stemness maintenance. In summary, taking the advantages of cross-linking density-mediated development of carbodiimide-treated gelatin matrices, a new niche can provide a significant boost to maintain the limbal stem cells during ex vivo expansion.

## Figures and Tables

**Table 1:** Ocular grading system used for biomicroscopic examinations.

Parameter	Ocular Score				
	0	1	2	3	4
Aqueous flare	Normal	Mild	Moderate	Severe	N/A ^1^
Anterior chamber fibrin	None	Mild	Moderate	Severe	N/A ^1^
Corneal cloudiness severity	Normal	Mild	Moderate	Severe	N/A ^1^
Corneal neovascularization	None	Mild	<180°	180°–360°	N/A ^1^
Iris neovascularization	None	Mild	<180°	180°–360°	N/A ^1^
Lens opacity	None	Mild	Moderate	Severe	N/A ^1^

^1^ Not applicable, because the biological responses are too severe to be observed.

**Table 2:** Sequences of primers used in gene expression analyses.

Genes ^1^	Forward (5′-3′)	Reverse (5′-3′)
Integrin β1	AGGAGAAAATGAATGCCAAATGG	GGGTGCTCATTTTCCCTCATACTT
ABCG2	GAGAGCTGGGTCTGGAAAAAGT	ATTCTTTTCAGGAGCAGAAGGA
GAPDH	TTGCCCTCAATGACCACTTTG	TTACTCCTTGGAGGCCATGTG

^1^ ABCG2: ATP binding cassette, subfamily G, member 2; GAPDH: glyceraldehyde-3-phosphate dehydrogenase.

## Supplementary Materials

Supplementary materials can be found at http://www.mdpi.com/1422-0067/19/11/3294/s1.
