# Supplementary material for: Effect of Cross-Linking Density on the Structures and Properties of Carbodiimide-Treated Gelatin Matrices as Limbal Stem Cell Niches
Source: Int J Mol Sci. 2018 Oct 23;19(11):3294. doi: 10.3390/ijms19113294 (PMC6274912; doi:10.3390/ijms19113294)

Supplementary Information

Figure S1. Schematic illustration of EDC cross-linking of gelatin.

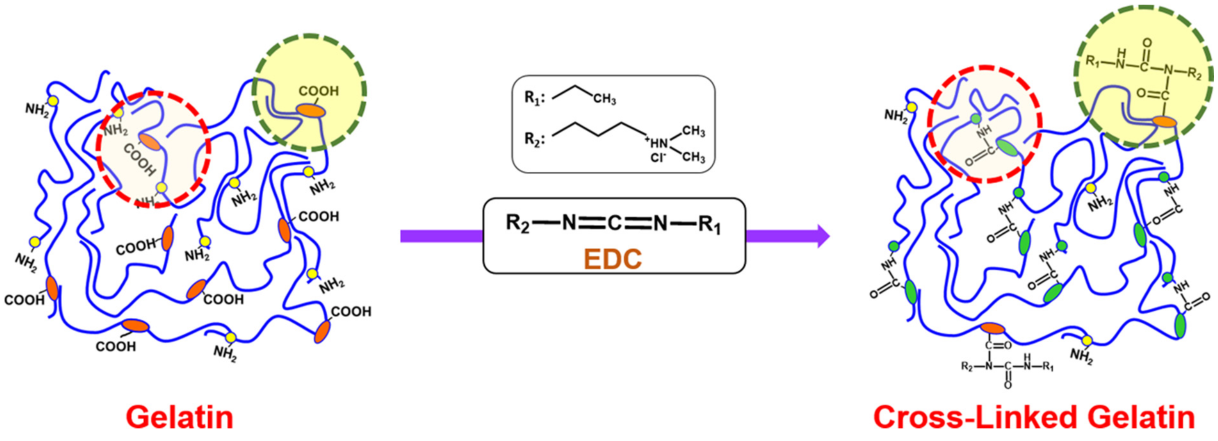

Figure S2. Phase-contrast micrographs of SIRC cultures after a 3-day exposure to gelatin samples cross-linked with 15 mM of EDC: (a) before and (b) after thorough washing of material samples with deionized water to remove unreacted EDC. Scale bars: 200 μm.

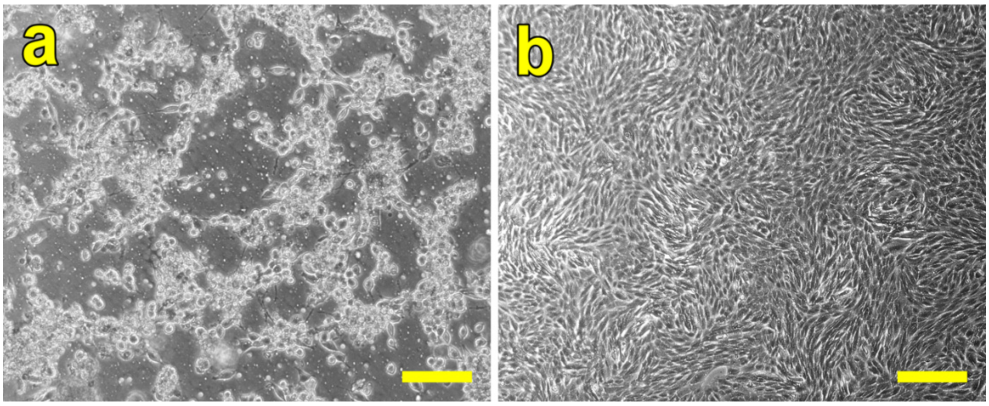

Supplement: Supplementary file 1 [file ijms-19-03294-s001.pdf]
